# Testing sensory drive speciation in cichlid fish: Linking light conditions to opsin expression, opsin genotype and female mate preference

**DOI:** 10.1111/jeb.13577

**Published:** 2019-12-24

**Authors:** Daniel Shane Wright, Roel van Eijk, Lisa Schuart, Ole Seehausen, Ton G. G. Groothuis, Martine E. Maan

**Affiliations:** ^1^ Groningen Institute for Evolutionary Life Sciences (GELIFES) University of Groningen Groningen The Netherlands; ^2^ University of Applied Sciences van Hall Larenstein Leeuwarden The Netherlands; ^3^ Institute of Ecology & Evolution University of Bern Bern Switzerland; ^4^ Department Fish Ecology & Evolution Eawag Center for Ecology, Evolution and Biogeochemistry Kastanienbaum Switzerland

**Keywords:** ecological speciation, Haplochromine, LWS, phenotypic plasticity, *Pundamilia*, visual pigment

## Abstract

Ecological speciation is facilitated when divergent adaptation has direct effects on selective mating. Divergent sensory adaptation could generate such direct effects, by mediating both ecological performance and mate selection. In aquatic environments, light attenuation creates distinct photic environments, generating divergent selection on visual systems. Consequently, divergent sensory drive has been implicated in the diversification of several fish species. Here, we experimentally test whether divergent visual adaptation explains the divergence of mate preferences in Haplochromine cichlids. Blue and red *Pundamilia* co‐occur across south‐eastern Lake Victoria. They inhabit different photic conditions and have distinct visual system properties. Previously, we documented that rearing fish under different light conditions influences female preference for blue versus red males. Here, we examine to what extent variation in female mate preference can be explained by variation in visual system properties, testing the causal link between visual perception and preference. We find that our experimental light manipulations influence opsin expression, suggesting a potential role for phenotypic plasticity in optimizing visual performance. However, variation in opsin expression does not explain species differences in female preference. Instead, female preference covaries with allelic variation in the long‐wavelength‐sensitive opsin gene (LWS), when assessed under broad‐spectrum light. Taken together, our study presents evidence for environmental plasticity in opsin expression and confirms the important role of colour perception in shaping female mate preferences in *Pundamilia.* However, it does not constitute unequivocal evidence for the direct effects of visual adaptation on assortative mating.

## Graphical abstract

*Pundamilia* cichlids exhibit light‐induced opsin expression plasticity, which correlates with female mate preference, but does not explain species differences in preference. Instead, female preference covaries with allelic variation in the LWS opsin in broad‐spectrum light, confirming the important role of colour perception in shaping *Pundamilia* mate preferences.

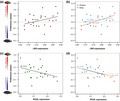

## 1 INTRODUCTION

Sensory drive—the hypothesis that sensory systems, signals and communication behaviour coevolve in concert with local environmental conditions (Endler, 1992)—has been implicated as a mechanism of divergence in a number of species. Many examples come from aquatic environments (Cummings & Endler, 2018), as the natural attenuation of light through water results in heterogeneous photic environments. Vision‐dependent aquatic species often possess visual systems, mating signals and mating behaviour correlated with the local light environment, implicating sensory drive‐like processes (e.g. guppies: Endler, 1992, sticklebacks: Reimchen, 1989; McDonald, Reimchen, & Hawryshyn, 1995; Boughman, 2001, 2002; Boughman, Rundle, & Schluter, 2005, killifish: Fuller, 2002; Fuller, Carleton, Fadool, Spady, & Travis, 2005; Fuller & Noa, 2010, swordtails: Kolm, Amcoff, Mann, & Arnqvist, 2012, surfperch: Cummings, 2007, pygmy perch: Morrongiello, Bond, Crook, & Wong, 2010, and cichlids: Maan, Hofker, Alphen, & Seehausen, 2006; Seehausen et al., 2008). Also in terrestrial systems, correlations between visual conditions and communication traits have been reported (Cummings, Bernal, Reynaga, Rand, & Ryan, 2008; Leal & Fleishman, 2004; McLean, Moussalli, & Stuart‐Fox, 2014).

When sensory adaptation directly affects not only ecological performance, but also traits related to sexual communication, assortative mating may evolve. In general, theory suggests that ecological speciation is facilitated when divergent adaptation immediately coincides with changes in mating patterns, such that individuals with the same adaptations mate among each other (Kirkpatrick & Ravigné, 2002). The traits that would mediate such a process have been labelled “magic”: powerful in driving fast speciation but assumed to be rare in nature (Gavrilets, 2004; Servedio, Doorn, Kopp, Frame, & Nosil, 2011; Smith, 1966). Sensory adaptation might function as a magic trait, mediating not only ecological adaptation, but also the detection and assessment of potential mates (Boughman, 2002; Maan & Seehausen, 2010). Alternatively, indirect selection, driven by variation in offspring fitness, may result in assortative mating among individuals with the same sensory adaptations: selection against recombinant offspring would favour the evolution of assortative mating preferences. This process relies on the build‐up and maintenance of linkage disequilibrium between the loci underlying sensory adaptation and mating preferences, and is much less efficient in generating reproductive isolation (Kirkpatrick & Barton, 1997; Maan & Seehausen, 2012; Servedio & Boughman, 2017). Here, we aim to establish whether divergent visual adaptation directly affects mating preferences in Lake Victoria cichlid fish.

*Pundamilia pundamilia* (Seehausen, Lippitsch, Bouton, & Heleen, 1998) and *Pundamilia nyererei* (Witte‐Maas & Witte, 1985) are two closely related species of cichlid fish found at rocky islands in south‐eastern Lake Victoria. Similar sympatric *Pundamilia* species pairs (*P. *sp.* “pundamilia‐like”* and *P. *sp.* “nyererei‐like”*) also occur at other rocky islands in south‐eastern portions of the lake (Meier, Marques, Wagner, Excoffier, & Seehausen, 2018; Meier et al., 2017). Males of the sympatric species are distinguished by their nuptial coloration; *P. pundamilia* and *P. *sp.* “pundamilia‐like”* are blue/grey, whereas *P. nyererei* and *P. *sp.* “nyererei‐like”* are orange/red dorsally and yellow on the flanks; all males have black, vertical bars on their flanks. Females are yellow/grey (Seehausen, 1996). The species pairs tend to be depth‐differentiated—the blue species is found in shallower waters, whereas the red species extends to greater depths (Seehausen, 1996; Seehausen et al., 2008). High turbidity in Lake Victoria results in a shift of the light spectrum towards longer wavelengths with increasing depth, such that the red species experiences very little short‐wavelength light (Castillo Cajas, Selz, Ripmeester, Seehausen, & Maan, 2012; Maan et al., 2006; Seehausen et al., 2008). Previous work has shown that male coloration mediates species‐assortative female preferences (Haesler & Seehausen, 2005; Seehausen & van Alphen, 1998; Selz, Pierotti, Maan, Schmid, & Seehausen, 2014; Stelkens et al., 2008).

Colour vision in cichlids (and vertebrates in general) is determined by photosensory pigments in the retina, comprised of a light‐sensitive chromophore bound to an opsin protein (Bowmaker, 1990). In *Pundamilia*, wild populations of blue and red species differ in the amino acid composition of the long‐wavelength‐sensitive opsin (LWS) (Seehausen et al., 2008) and behavioural tests revealed that *P. nyererei* is more sensitive to long‐wavelength (red) light and *P. pundamilia* is more sensitive to short‐wavelength (blue) light (Maan et al., 2006). Correspondence between these factors—differences in the photic environment, visual system properties, male coloration and female colour preference—suggests that sensory drive contributes to the divergence of these two species (Maan & Seehausen, 2010). However, experimental tests are required to establish a causal relationship between visual perception and mate preference.

In addition to opsin allelic variation, visual sensitivity is determined by differential usage of vitamin A1‐ versus. A2‐based chromophores and the expression levels of the opsin genes (Carleton, 2009). Light‐induced changes in opsin expression have been observed in several fish species, including cichlids (Dalton, Lu, Leips, Cronin, & Carleton, 2015; Fuller & Claricoates, 2011; Fuller, Noa, & Strellner, 2010; Hofmann, O'Quin, Smith, & Carleton, 2010; Nandamuri, Yourick, & Carleton, 2017; Shand et al., 2008; Smith, Ma, Soares, & Carleton, 2012; Stieb, Carleton, Cortesi, Marshall, & Salzburger, 2016; Van der Meer, 1993; Veen, Brock, Rennison, & Bolnick, 2017). This provides an experimental opportunity to manipulate visual system development. Here, we aim to experimentally induce variation in opsin expression and test its consequences for female mate choice. Thus, we aim to induce a plastic response to mimic the effects of visual adaptation. We recreated the shallow‐water and deep‐water light environments of Lake Victoria, and reared each species in both light conditions. In a prior study, we found that these manipulations influenced female mate preference: shallow‐reared females (broad‐spectrum light) preferred blue males, whereas deep‐reared females (red‐shifted light) tended to prefer red males (Wright et al., 2017). This was not due to changes in male colour signalling, as nuptial colour (blue/red) was unaffected by our light manipulations (Wright, Rietveld, & Maan, 2018). Here, we investigate whether the observed change in female preference can be ascribed to variation in opsin expression. We also test whether female preference covaries with allelic variation in the LWS gene.

## 2 METHODS

### 2.1 Experimental fish

F1 offspring of wild‐caught *P. *sp.* “pundamilia‐like”* and *P. *sp.* “nyererei‐like”* (hereafter referred to as the blue or red species, respectively), collected in 2010 and 2014 at Python Islands (−2.6237, 32.8567) in the Mwanza Gulf of Lake Victoria, were reared in manipulated light environments mimicking the shallow and deep waters at Python Islands (described in detail below). Fish collected in 2010 were first transported to the Eawag Institute at Kastanienbaum, Switzerland; in September 2011, they were relocated to the University of Groningen, the Netherlands. Light conditions were the same for both locations—described in detail below. F1 families (hybrid and nonhybrid) were created opportunistically as reciprocal crosses, with 25 dams and 20 sires. Thirty‐two crosses (11 red × red; 7 blue × blue; 7 red × blue; 7 blue × red) resulted in a test population of 85 fish from 30 families (2 red × red and 2 red × blue crosses were full‐sibs; family details provided in Table S1). We included hybrids because their heterozygosity (particularly at loci influencing visual properties and mate preference) could allow the manifestation of environment‐induced effects, which may be obscured by strong genetic effects in the parental species. Hybridization occurs at low frequency at Python Islands (Seehausen et al., 2008) and can be accomplished in the laboratory by housing females with heterospecific males. *Pundamilia* are maternal mouthbrooders; to reduce the opportunity for imprinting (Verzijden & ten Cate, 2007), fertilized eggs were removed from brooding females approximately 6 days after spawning (mean ± se: 6.3 ± 0.5 days post‐fertilization; eggs hatch at about 5–6 dpf) and split evenly between light conditions. Fish were maintained at 25 ± 1°C on a 12L: 12D light cycle and fed daily a mixture of commercial cichlid flakes, pellets and frozen food (artemia, krill, spirulina, black and red mosquito larvae). This study was conducted under the approval of the Veterinary Office of Kanton Lucerne (01/10) and the Institutional Animal Care and Use Committee of the University of Groningen (DEC 6205B; AVD105002016464).

### 2.2 Experimental light conditions

Our manipulated light conditions were created to mimic the natural light environments of the blue and red species at Python Islands, Lake Victoria (described in greater detail in: Maan, Seehausen, & Groothuis, 2017). Briefly, we used halogen light bulbs filtered with a green light filter (LEE # 243, Andover, UK) in both conditions. In the “shallow” condition, mimicking the blue species' habitat, the spectrum was supplemented with blue light (*Paulmann* 88090). In the “deep” condition, mimicking the red species' habitat, short‐wavelength light was reduced by adding a yellow light filter (LEE # 015). Our light conditions were designed to mimic in particular the spectral differences between habitats, and only partly recreated depth differences in light intensity (the deep condition had a light intensity of ~70% of that of the shallow condition; at Python Islands, light intensity in the deep environment (measured in 2010) was ~35% of that in the shallow environment; Figure S1).

### 2.3 Female preference assay

The mate preference data came from a prior study of 91 females (Wright et al., 2017), conducted from May 2012 to September 2014 at the University of Groningen. In short, we used a dual‐choice preference design; for each trial, a randomly chosen, sexually mature, gravid female (>6 months age) was introduced into the centre portion of a large tank and allowed to interact with males housed on opposite ends of the tank. The males (one of each species: blue vs. red) were confined behind transparent barriers, with a PVC tube and stone for shelter. We scored male courtship behaviour—lateral display and quiver (McElroy & Kornfield, 1990), the first two behaviours in the sequence of the haplochromine courtship ritual (Seehausen, 1996)—and the corresponding female response to each male courtship event (positive or negative). As in prior studies of *Pundamilia* (Haesler & Seehausen, 2005; Maan et al., 2004; Seehausen & van Alphen, 1998), positive female response was classified by an observable interest in male behaviour—moving towards males and/or remaining engaged in interaction (i.e. still trying to gain access to the male through the plastic partition following male courtship). Negative responses were classified as a general disinterest—moving away and/or not responding to male behaviour. All females were PIT‐tagged (Passive Integrated Transponders, from Biomark, Idaho, USA, and Dorset Identification, Aalten, the Netherlands), allowing blind behaviour scoring, and tested repeatedly under both shallow and deep light conditions, with different combinations of size‐matched stimulus males. Behavioural scoring started when females entered a male interaction zone (20 cm in front of each male) and was paused when females left this zone, until a total of 20 min of interaction time (combined across the two zones) was reached. Trials were considered successful if 20 min of interaction time was recorded within one hour and each male had performed at least three quiver displays.

Female positive and negative responses to each male courtship behaviour were totalled for each trial, separately for lateral display (LD) and quiver (Q), and female preference scores were calculated as the difference in the proportions of positive responses to male courtship between the two males. For example, LD‐based preference was calculated as follows:

$$\text{Preference LD}\;=\;\left(\frac{\text{Positive to red male LD}}{\text{Total red male LD}}\right)-\left(\frac{\text{Positive to blue male LD}}{\text{Total blue male LD}}\right)$$

The result is a measure of preference ranging from −1 to 1, with positive scores indicating a preference for red males and negative scores indicating a preference for blue males. Q‐based preference was calculated in the same way.

### 2.4 Opsin mRNA expression

Cichlids possess seven distinct classes of opsins, one rod opsin (RH1—functions in low light) and six cone opsins that mediate colour vision in bright light. The cichlid cone opsins include (Carleton et al., 2008) the short‐wavelength‐sensitive opsins: SWS1 (UV), SWS2b (violet) and SWS2a (blue), the rhodopsin‐like opsins: RH2B, RH2Aβ and RH2Aα (green), and the long‐wavelength‐sensitive opsin: LWS (red). Typically, cichlids express a subset of three cone opsins at a time, the relative proportions of which influence colour vision (Carleton, 2009). In Lake Victoria, all seven species studied so far express SWS2a, RH2A and LWS (and low amounts of SWS2b: Hofmann et al., 2009).

For opsin expression, laboratory‐bred fish were sacrificed  with an overdose of MS‐222 and the eyes extracted and preserved in *RNAlater*™ (Ambion). Mean (± *SE*) age at sampling was 829.2 ± 44.6 days (min/max = 186/1827 days). To maximize RNA yield and minimize differences due to circadian variation in opsin expression (Halstenberg et al., 2005), all fish were euthanized  at approximately the same time in the early evening (~16:00–18:00, *n* = 59 fish). A smaller number of fish (*n* = 17) were sampled opportunistically, from individuals that died for reasons unrelated to the experiment (e.g. aggression). Information on sample type (euthanized vs. found dead) was not recorded for 9 fish. In total, we sampled 37 males (14 from deep, 23 from shallow) and 38 females (18 from deep, 20 from shallow); sex was not recorded for 10 fish (three from deep, seven from shallow). Twenty‐five (of the 38) females, derived from 14 dams (five red and nine blue) and nine sires (five red and four blue; see Table S3), were previously tested for mate preference (Wright et al., 2017).

We used real‐time polymerase chain reaction (qPCR) to determine the relative amount of each cone opsin gene expressed (Wright et al., 2019). From preserved eyes, we removed the retina and isolated total RNA using TRIzol (Ambion). We reverse‐transcribed one microgram of total RNA using oligo(dT)_18_ primer (Thermo Scientific) and RevertAid H Minus Reverse Transcriptase (Thermo Scientific) at 45°C to create retinal cDNA. qPCRs were set up for each of the four cone opsins expressed in *Pundamilia* (SWS2b, SWS2a, RH2A and LWS) using TaqMan chemistry (Applied Biosystems) and gene‐specific primers and probes (Table S2). As in previous studies, we collectively measured the functionally and genetically similar RH2Aα and RH2Aβ as RH2A (Carleton, Parry, Bowmaker, Hunt, & Seehausen, 2005; Carleton et al., 2008; Hofmann et al., 2009; Spady et al., 2006). Fluorescence was monitored with a CFX96 Real‐Time PCR Detection System (Bio‐Rad) over 50 cycles (95°C for 2 min; 95°C for 15 s; 60°C for 1 min).

We used *LinRegPCR* (Ramakers, Ruijter, Deprez, & Moorman, 2003) to determine the critical threshold cycle numbers (*C_t_*) for all four opsin genes. This approach examines the log‐linear part of the PCR curve for each sample, determining the upper and lower limits of a “window‐of‐linearity” (Ramakers et al., 2003). Linear regression analysis can then be used to calculate the individual PCR efficiency and to estimate the initial concentration (*N*_0_) from a line that best fits the data (Ramakers et al., 2003). In this way, *N*_0_ values can be estimated without having to assume equal PCR efficiencies between amplicons (Ramakers et al., 2003). All samples were run in duplicate, and for consistency, we applied specific quality control parameters: PCR efficiency 75% – 125% and Ct standard deviation ≤0.5. We used the mean of the duplicate *N*_0 _estimates to calculate relative expression levels for each sample (described below).

On each plate, we included a serially diluted construct containing one fragment of each of the four opsin genes ligated together. From this, we used linear regression to examine the relationship between log (concentration) and Ct values of the construct, enabling us to calculate the slope (*m*) and intercept (*b*) of the regression. Using these values, we calculated relative cone opsin expression as:

$$\frac{N_{0i}}{N_{0\text{all}}}\;=\;\frac{\exp^{\frac{\left({\text{ct}}_{i}-b\right)}{m}}}{\sum \;\exp^{\frac{\left({\text{ct}}_{i}-b\right)}{m}}}$$

where *N*_0_ *_i_/N*_0all_ is the expression for a given opsin gene relative to the total expression of all measured opsin genes, Ct*_i_* is the critical threshold value for the focal sample, and *b* and *m* are the intercept and slope values derived from the construct linear regression (as detailed in: Gallup, 2011).

### 2.5 LWS sequence variation

We sequenced the LWS gene of females previously assessed for mate preference (Wright et al., 2017). *Pundamilia* from Python Island harbour two forms of the LWS gene: the “H” allele, with peak sensitivity at 559 ± 1 nm, and the “P” allele, with peak sensitivity at 544 ± 3 nm (Seehausen et al., 2008). The “H” allele occurs predominantly in *P. *sp.* “nyererei‐like”*, whereas the “P” allele occurs predominantly in *P. *sp.* “pundamilia‐like”*, but hybridization results in a small number of “mismatched” allele types (e.g. *P. *sp.* “nyererei‐like”* that are heterozygous or “PP”). The two alleles differ in only three amino acid positions (216, 230, 275), located on the fourth and fifth exons (Seehausen et al., 2008; Terai et al., 2006). From fin clips, we isolated DNA (Meeker, Hutchinson, Ho, & Trede, 2007) and sequenced (*Sanger* sequencing, GATC Biotech) exons 4 and 5 (498 bp, including the 91‐bp intron; forward primer: GTTTGGTGTGCTCCTCCCAT; reverse primer: CAGAGCCATCGTCCACCTGT; see also Figure S2). We categorized individuals as “H” if: 216Y, 230A, 275C and “P” if: 216F, 230T, 275I (as in: Seehausen et al., 2008; Wright et al., 2019). All fish were sequenced twice, in forward and reverse directions, and alignments were performed in Mega 7 (Kumar, Stecher, & Tamura, 2016), using the LWS coding sequences reported in Seehausen et al. (2008) as reference. For 35 individuals, we observed multiple peaks at one or more of the polymorphic nucleotide sites (see Figure S3), so we also categorized fish as “heterozygous”. In total, we sequenced 65 females (Table S4), allowing us to assign LWS genotype to 77 (using pedigree information). Twenty‐four of these females were also measured for opsin expression (we were unable to genotype one of the 25 females that we had mate preference and opsin expression data for; Table S3).

### 2.6 Statistical analyses

#### 2.6.1 Variation in opsin expression

Prior to analyses, expression data were filtered for outliers, that is values outside 1.5 * the interquartile range (IQR). This was done separately for each combination of opsin/species/rearing light treatment, resulting in 85 samples (28 removed by filtering). The relative expression of each opsin is defined in relation to the other three opsins, so discarding a sample for one opsin meant the entire sample was discarded (22 of 28 samples were removed because of one opsin). We used this additional filtering step to ensure that (a) all data were consistently within a natural range of expression values (as documented in: Carleton et al., 2005; Hofmann et al., 2009; plus our own measurements of opsin expression in wild fish; Wright et al., 2019) and (b) was not influenced by tissue quality (fish sacrificed  vs. found dead).

Using linear mixed modelling (*lmer* function in the *lme4* package: Bates, Maechler, Bolker, & Walker, 2014), we explored how opsin expression was influenced by the effects (and interactions) of rearing light (deep vs. shallow), species (blue, red, hybrid) and sex (male vs. female) as: expression~light*species*sex. Random effects included maternal and paternal identity and age to account for: (a) shared parentage among sampled fish (see Table S1) and (b) age differences at sampling (all fish were sexually mature adults but age ranged from 183 to 2,601 days). The optimal random effect structure was determined by AIC comparison (Sakamoto, Ishiguro, & Kitagawa, 1986), and the significance of fixed effect parameters was determined by likelihood‐ratio tests (LRT) via the *drop1* function. Minimum adequate statistical models (MAM) were selected using statistical significance (Crawley, 2002; Nakagawa & Cuthill, 2007). We used the ANOVA function in the *car* package (Fox et al., 2017) to estimate the parameters of significant fixed effects, with Kenward–Roger degrees of freedom (Halekoh & Højsgaard, 2014; Kenward & Roger, 1997). In the case of more than two categories per fixed effect parameter (i.e. species), we used the post hoc Tukey tests (glht—multcomp package: Hothorn, Bretz, & Westfall, 2008) to obtain parameter estimates and report P‐values adjusted for multiple comparisons.

#### 2.6.2 Relationship between female preference and visual properties

To examine the relationship between opsin expression and female preference behaviour, we used the same linear mixed modelling approach described above for the subsample of females measured for opsin expression (Table S3). Thus, we tested: female preference~expression*rearing light*test light. Random effects included the following: female identity, male identity, parental identity and observer identity to account for: (a) repeated testing of females, (b) repeated usage of stimulus males, (c) shared parentage among test females (Table S1), and (d) multiple scorers of behaviour.

We also examined the correlation between LWS allelic variation and female preference behaviour as: preference~genotype*rearing light*test light. Our data set was not large enough to include the interaction of all four variables in the same model (opsin expression, LWS genotype, rearing light and testing light), so we examined the combined influence of both visual properties in a simplified model: preference~expression*genotype.

## 3 RESULTS

### 3.1 Species differences in opsin expression

**Figure 1:**
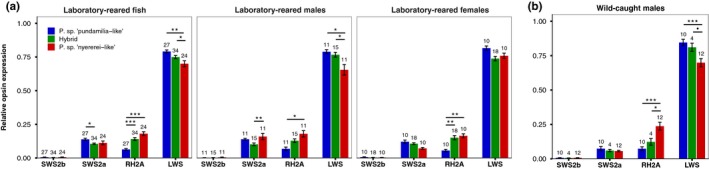
Opsin expression—(a) Opsin expression profiles of laboratory‐reared fish, showing higher LWS and lower RH2A expression in blue fish compared to red fish. These patterns closely mimicked those of (b) wild‐caught males from Python Island. Opsin expression data for wild fish are from Wright et al., 2019, and based on males only; females were not sampled because they are difficult to identify with certainty in the field. Sample sizes are given above each bar, and error bars represent ± standard error. ***indicates *p* < .001, **indicates *p* < .01, *indicates *p* < .05, • indicates *p* < .1

Independent of our light treatments, we found species differences in opsin expression. LWS expression differed significantly among the species (*F*_2,18.25_ = 5.07, *p* = .017; Figure 1a). Contrary to expectation, LWS expression was highest in the blue species (Tukey's post hoc blue vs. red: *Z* = 3.50, *p* = .001, blue vs. hybrid: *p* = .25) and lowest in the red species (vs. hybrids: *Z* = −2.62, *p* = .023). RH2A expression also differed (*F*_2,23.08_ = 12.43, *p* < .001; Figure 1a); post hoc showed that it was lowest in the blue species, differing significantly from the red species (*Z* = −5.39, *p* < .001) and hybrids (*Z* = −4.74, *p* < .001). The red species and hybrids did not differ in RH2A expression (*p* = .12). A weak trend indicated species differences in SWS2a expression (*F*_2,16.92_ = 2.71, *p* = .095); post hoc revealed higher SWS2a expression in the blue species compared to hybrids (*Z* = 2.52, *p* = .03), but all other comparisons were nonsignificant (*p* > .3). Finally, SWS2b expression did not differ among species (*p* = .44). These observations closely resemble the species‐specific expression patterns of wild‐caught males from the same location (Figure 1b).

### 3.2 Sex‐specific opsin expression

**Figure 2:**
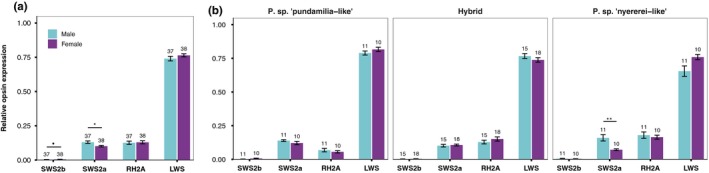
Sex‐specific opsin expression—The expression of the short‐wavelength‐sensitive opsins differed between the sexes: males expressed more SWS2a, and females tended to express more SWS2b. (a) All fish; (b) separated by species. Sample sizes are indicated above each bar, and error bars represent ± standard error. *indicates *p* < .05, • indicates *p* < .1

We also found differences in opsin expression between males and females, but only for the short‐wavelength‐sensitive opsins (Figure 2a). For SWS2a, we found an interaction of species and sex (*F*_2,34.21_ = 3.69, *p* = .035); post hoc tests showed that red males expressed more SWS2a than red females (*Z* = 3.93, *p* = .0012, Figure 2b) although there were no sex differences in the blue species or in the hybrids (*p* > .9). Overall, independent of species, males expressed more SWS2a than females (*F*_1,54.99_ = 4.72, *p* = .034), whereas females tended to express more SWS2b than males (*F*_1,65.57_ = 3.72, *p* = .057). There were no sex differences in RH2A or LWS expression (*p* = .44 and *p* = .73, respectively). The reported species differences (above) and light‐induced effects (below) are independent of sex (sex was included as a covariate in all models).

### 3.3 Rearing light influences opsin expression

**Figure 3:**
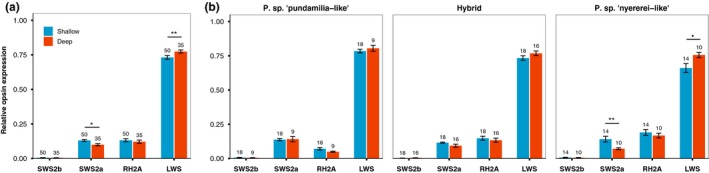
Light‐induced changes in opsin expression—(a) The relative expression of long (LWS) and short‐wavelength (SWS2a) opsins was significantly influenced by our light manipulations. (b) *P*. sp. “*nyererei‐like*” was most strongly influenced. Sample sizes are indicated above each bar, and error bars represent ± standard error. **indicates *p* < .01, *indicates *p* < .05, and • indicates *p* < .1

Rearing light significantly influenced opsin expression (Figure 3a): deep‐reared fish had higher LWS expression (*F*_1,64.86_ = 7.53, *p* = .007) and lower SWS2a expression (*F*_1,55.87_ = 6.99, *p* = .01) than shallow‐reared fish. RH2A (*p* = .38) and SWS2b (*p* = .39) were not influenced by rearing light. For SWS2b, we found a significant interaction between rearing light and species (*F*_2,53.69_ = 3.49, *p* = .038), though Tukey's post hoc showed no differences for any of the pairwise between‐treatment, within‐species comparisons (*p* > .43). For the other three opsins, we found no interactions between rearing light and species, indicating similar responses across species (*p* > .15). However, Figure 3b suggests stronger effects of the rearing environment in *P. *sp.* “nyererei‐like”*. Tukey's post hoc supported this: deep‐reared *P. *sp.* “nyererei‐like”* had lower SWS2a expression (*Z* = −3.69, *p* = .002) and tended to have higher LWS expression (*Z* = 2.82, *p* = .051), in comparison with their shallow‐reared counterparts. Together, these results show that our light manipulations significantly influenced patterns of opsin expression, especially in *P. *sp.* “nyererei‐like”*.

### 3.4 Does female mate preference covary with opsin expression?

A subset of females (*n* = 25) tested for mate preference (Wright et al., 2017) allowed us to explore the link between light‐induced variation in opsin expression and variation in female preference behaviour. Opsin expression, as an individual effect, never influenced female preference (*p* > .21 for both preference measures; for all females combined). This was also true for each female species group separately (blue: *p* > .36; red: *p* > .18; hybrid: *p* > .44). The fact that females were tested under different light conditions did not impact this result; there was no effect of test light (*p* > .42).

**Figure 4:**
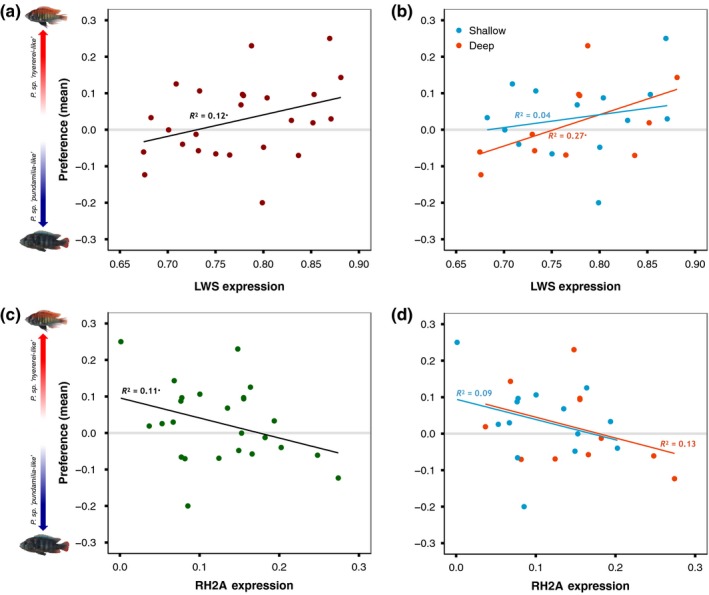
Opsin expression covaries with female preference—Mean female preference had a weak, positive relationship with (a) LWS expression and a weak, negative relationship with (c) RH2A expression. (b & d) Similar relationships were exhibited by both deep‐ and shallow‐reared females, indicating that the relationship between preference and expression was not due to the light manipulations. • indicates *p* < .1

The repeatability of individual preference behaviour in our prior study was low (R_LD_ = 0.103; R_Q_ = 0.07; females were tested multiple times; see Figure S4); thus, subtle relationships between female preference and opsin expression may have been masked by within‐female variation. Therefore, we also calculated mean preference scores for each female and repeated the analyses. This yielded a weak positive relationship between LWS expression and mean quiver preference (*r* = 0.356, *df* = 23, *p* = .085; Figure 4a) and a weak negative relationship between RH2A expression and mean quiver preference (*r* = −0.341, *df* = 23, *p* = .094; Figure 4c). Importantly, these relationships were not caused by the light manipulation: similar trends were present in both deep‐ and shallow‐reared females (Figure 4b,d; the interactions of rearing light and LWS/RH2A expression were both nonsignificant, *p* > .45). A causal effect of the light manipulation would be evidenced by a shift along the *y*‐axis (the intercepts of the slopes for deep‐reared females should have been higher than the intercepts of the slopes for shallow‐reared females; they were not). Moreover, the two relationships are opposite to those observed *across* the blue and red species: higher LWS expression and lower RH2 expression are characteristic for blue rather than red females (see above) but are associated with preference for red males in the experimental females. This implies that these trends do not explain the species difference in preference. Finally, preference did not covary with expression of SWS2b or SWS2a (*p* > .17).

### 3.5 Distribution of LWS genotypes

**Figure 5:**
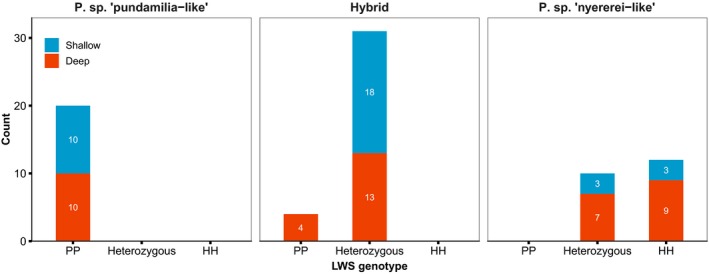
Distribution of LWS genotypes between species and light treatments—LWS genotypes for 77 females. Sample sizes are indicated in each bar

Of the 91 females tested for preference behaviour (Wright et al., 2017), we were able to assign LWS genotype to 77 (Figure 5). All blue females (both parents blue) were homozygous “PP” (*n* = 20). Within the red females (both parents red), twelve were “HH” but ten were heterozygous. Thirty‐one hybrid females were heterozygous, whereas four hybrids (all with blue dam, red sire) were homozygous “PP”. Genotypes were distributed equally between both light treatments (Figure 5). Opsin expression for each genotype/species combination is presented in the supplementary information (Figure S5).

### 3.6 Does female mate preference covary with LWS genotype?

**Figure 6:**
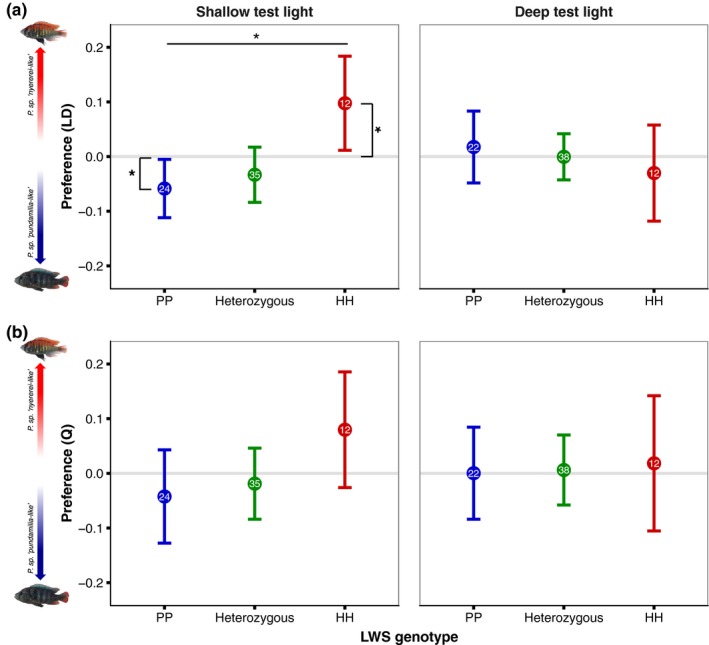
Association between LWS genotype and preference—Mean female (LD) preference was influenced by an interaction of test light and LWS genotype. In shallow test light, “PP” females preferred blue males and “HH” females preferred red males. When tested in deep light, there was no difference between genotypes. Sample sizes are given within each circle, and error bars represent 95% C.I. *indicates *p* < .05

LWS genotype did not covary with preference (*p* > .41 for both preference measures; also true for mean preference scores, *p* > .12). However, there was a difference between test light conditions: female preference (LD) was influenced by an interaction between LWS genotype and test light (*F*_2,201.47_ = 4.79, *p* = .009; Figure 6a). Tukey's post hoc revealed a significant difference between “HH” and “PP” genotypes when tested in shallow light (*Z* = 2.89, *p* = .041): “PP” females preferred blue males (the intercept differed significantly from zero; 95% CI [−0.121, −0.007]), whereas “HH” females preferred red males (95% CI [0.0004, 0.1809]). All other comparisons, including those from deep test light, were nonsignificant (*p* > .17). Quiver preference was unaffected (the same interaction was nonsignificant, *p* = .52), though the trends were similar (Figure 6b).

At Python Island, LWS genotype is nearly fixed in each species (Seehausen et al., 2008). Thus, the interaction between LWS genotype and test light may be due to other species‐specific genetic factors. We tested the influence of species * test light on female preference (we had several combinations of LWS genotype and species identity among our tested females; see Figure 3) but found that the interaction was nonsignificant (LD: *p* = .15, Q: *p* = .9; Figure S6). We also analysed the combined effects of LWS genotype and opsin expression on preference, but again found no significant interaction effects (all *p* > .32). Together, these results may indicate that LWS genotype, more than opsin expression or other species‐specific genetic factors, contributes to species differences in female preference.

## 4 DISCUSSION

Sensory drive, the hypothesis that sensory perception, communication signals and behaviour coevolve in concert with the local environment, has been implicated as a diversifying mechanism in several fish species. To experimentally test for a causal relationship between species differences in visual perception and mate preference, we reared two species of Lake Victoria cichlids—*P. *sp.* “pundamilia‐like”* and *P. *sp.* “nyererei‐like”*—in light environments resembling the shallow and deep photic conditions of Lake Victoria. In this way, we aimed to induce changes in opsin expression, thereby mimicking aspects of divergent visual adaptation. We had previously shown that these light manipulations influence female mate preference, and here, we examined to what extent this can be attributed to changes in opsin expression.

### 4.1 Environmental light influences opsin expression

Our results show that relative opsin expression is influenced by the light environment experienced during development. In particular, it is the opsins at either end of the light spectrum that are affected: deep‐reared fish expressed more LWS, and shallow‐reared fish expressed more SWS2a. This follows previous work showing plasticity in cichlid visual development (Hofmann et al., 2010; Nandamuri et al., 2017; Smith et al., 2012; Van der Meer, 1993). In contrast with prior studies, however, our light manipulations were relatively subtle, mimicking the natural spectral (and partly intensity) differences in Lake Victoria. More extreme light conditions (in either spectra or intensity) would probably induce even greater changes in opsin expression and could be used to explore scenarios of future environmental change or range expansion (e.g. to greater depths). Our results are also consistent with the expression patterns of wild‐caught fish (see Figure 1b).

When examining species‐specific responses to the light manipulations, we found stronger effects in the red species, again at the spectrum extremes. Shallow‐reared *P. *sp.* “nyererei‐like”* expressed more SWS2a and tended to express less LWS, whereas *P. *sp.* “pundamilia‐like”* and hybrids did not differ significantly between light conditions. This suggests that opsin expression in the red species is more plastic. Seehausen et al. (2008) reported that the depth range of *P. *sp.* “nyererei‐like”* at Python Island is 0–5 m, whereas *P. *sp.* “pundamilia‐like”* occurs no deeper than 2 metres. Thus, opsin plasticity in the red phenotypes could be related to the fact that they naturally experience a wider range of light environments (as shown in figure 4 in: Castillo Cajas et al., 2012). It is also possible that plasticity in opsin expression contributed to the origin of the red species: individuals with greater visual plasticity might have been more likely to colonize and persist in the deeper waters not exploited by the blue phenotypes. Other studies have also reported differences in the plasticity of opsin expression between different cichlid species (Hofmann et al., 2010; Nandamuri et al., 2017) and shown that experimentally induced variation in LWS expression influences cichlid visual sensitivity to red stimuli (Smith et al., 2012). Together, these studies suggest a broader role for visual plasticity in cichlid visual adaptation and speciation. This plasticity may aid visual performance in a new or changing environment, for example when fish move between habitats (i.e. depths or different locations) or when confronted with environmental change (either natural or human‐induced). How and to what extent opsin expression plasticity contributes to visual performance in a new environment is the subject of ongoing work.

### 4.2 Opsin expression is weakly correlated with female preference

To test for a causal link between changes in opsin expression and female preference behaviour, we used 25 females that were each tested multiple times for blue‐red preference. Based on prior work (Carleton et al., 2005; Hofmann et al., 2009), we expected that, across *Pundamilia* populations, the red species expressed more LWS and the blue species more SWS. On this assumption, we designed this experiment to manipulate opsin expression and test its effect on female mate preference. We now know that in our study population (Python Island), the blue species expresses more LWS than the red species (see Figure 1 and Wright et al., 2019), implying a mismatch between species‐specific opsin expression and species‐specific preference: high LWS expression, presumably causing greater red sensitivity, is associated with preference for blue males. Correspondingly, we found no relationship between individual‐level preferences and opsin expression.

When considering mean preference scores per female, we found weak correlations for both LWS expression and RH2A expression, indicating that opsin expression may influence female preference behaviour. However, this does not explain the species difference in preference. This is because the relationships are opposite to those observed *across* the two species: high LWS and low RH2A expression, associated with preference for red males in the experimental females, are characteristic of the blue species. This suggests a possible influence of relative opsin expression on female preference that is not caused by other species‐specific factors that are linked to opsin expression. If it were, such species‐specific factors should have resulted in a negative correlation between LWS expression and female preference for red males. In line with this, the preference–expression relationship was not influenced by species identity or LWS genotype (see Figure S7). Importantly, deep‐ and shallow‐reared females displayed similar relationships between expression and preference, in both intercept and slope. This implies that, although rearing light influenced both female preference (shallow‐reared females preferred blue males, and deep‐reared females preferred red males; Wright et al., 2017) and opsin expression (deep‐reared fish expressed more LWS and less SWS2a), evidence for a causal link between opsin expression and preference is lacking. Thus, we do not find support for the hypothesis that variation in opsin expression serves as a “magic trait” in *Pundamilia* speciation, pleiotropically affecting both visual perception and mate choice.

This, of course, does not mean that opsin expression has no influence on female preference behaviour at the individual level; our results suggest that it might. Additional work is needed to explore this further, as our findings are based on a small sample size—we had opsin expression and mate preference data for only 25 females. This is enough to conclude that differences in opsin expression do not explain the species difference in female mate preference, but not to explore individual variation *within* species.

### 4.3 Female preference covaries with LWS genotype

This study was specifically designed to test the causal link between opsin expression and female mate preference. To test the contribution of LWS genotype to mate preference would have required a larger test population of females with segregating alleles. Given the importance of LWS allelic variation in these species, we also genotyped the females tested for mate preference. We found an environment‐dependent relationship between LWS genotype and female preference (see Figure 6 and Figure S6). When tested in broad‐spectrum light, “HH” females (all *P. *sp.* “nyererei‐like”*) preferred red males, whereas “PP” females (predominantly *P. *sp.* “pundamilia‐like”*) preferred blue males. LWS is nearly fixed in each species at Python Island (Seehausen et al., 2008), and prior work in *Pundamilia* has also documented species‐assortative female preferences for male colour in broad‐spectrum light (Haesler & Seehausen, 2005; Seehausen & van Alphen, 1998; Selz et al., 2014). However, the prior studies did not consider LWS genotype. Thus, our results may be due to two factors: 1) LWS genotype is causally linked to visual perception and preference determination or 2) the variation we observe is due to other species‐specific factors unrelated to visual perception. In our sample, LWS genotype was not synonymous with female species identity—there were 10 red‐type females that were heterozygous for LWS and 4 hybrids that were homozygous “PP” (Figure 3). We tested the influence of species * test light on female preference, but the interaction was nonsignificant, in contrast to the LWS * test light interaction (see Figure S6). This suggests that the latter is not driven by species‐specific preference loci unrelated to visual perception. These results are, of course, correlational; to confirm a role for LWS, future studies should directly target and manipulate LWS genotypes.

### 4.4 Low repeatability in female preference behaviour

Opsin expression was (weakly) correlated with mean preference scores but not with individual‐level preference scores (see above). In addition to the small sample size, we attribute this discrepancy to the low repeatability in female preference, which may have masked a relatively subtle relationship with opsin expression. Previous work in *Pundamilia* reported higher female preference repeatability (R = 0.59; Haesler & Seehausen, 2005), but this was for females reared and tested under white light. Females in our study were reared and tested in manipulated light conditions; the spectra of our light treatments mimicked natural conditions but differed dramatically from the standard aquarium lighting (see Figure S1). This may have influenced repeatability scores. Also in contrast to prior work (Dijkstra, Zee, & Groothuis, 2008; Haesler & Seehausen, 2005; Seehausen & van Alphen, 1998; van der Sluijs et al., 2008), we limited the opportunity for maternal imprinting (Verzijden & ten Cate, 2007; Verzijden, Korthof, & Cate, 2008) by removing fish from brooding females at 5 – 6 days post‐fertilization, possibly reducing preference strength. Finally, we are now aware of light‐induced survival differences in our laboratory population: when reared in “unnatural” light conditions, while all other parameters are kept the same, both species survive at a lower rate (~40% reduction at 12 months) than their “naturally” reared counterparts (Maan et al., 2017). Nonrandom survival could have generated a population of relatively flexible test females, exhibiting weak species specificity in behaviour including mate choice. It is also possible that our experimental design poorly estimated mate choice. This seems unlikely, however: pilot studies suggest that the preference scores measured in our experimental set‐up do predict subsequent spawning decisions (see Figure S8).

### 4.5 Sexually dimorphic opsin expression

We found that males had higher SWS2a expression, whereas females tended to express more SWS2b (Figure 2a). These patterns were largely independent of our light treatments (discussed above) and were consistent between *P. *sp.* “pundamilia‐like”* and *P. *sp.* “nyererei‐like”* (but perhaps more pronounced in *P. *sp.* “nyererei‐like”*). Sex differences in opsin expression have been observed in other taxa, for example butterflies (Arikawa, 2005; Everett, Tong, Briscoe, & Monteiro, 2012; McCulloch, Osorio, & Briscoe, 2016; Sison‐Mangus, 2006) and birds (Bloch, 2015), but we are aware of only one example in fish (guppies; Laver & Taylor, 2011) and none in cichlids. Possibly, these observed differences are related to ecological differences between the sexes: males defend territories at the lake bottom, whereas females of *P. *sp.* “nyererei‐like”* often shoal in the water column (Seehausen, 1996). It is possible that higher SWS2b expression helps females forage on small prey items; in sticklebacks (Rick, Bloemker, & Bakker, 2012) and Lake Malawi cichlids (Hofmann et al., 2009; Jordan, Howe, Juanes, Stauffer, & Loew, 2004), UV vision contributes to foraging performance. Given the novelty of this result, sexually dimorphic opsin expression in cichlids deserves more attention.

## 5 CONCLUSION

In this study, we aimed to explore the causal relationship between divergent visual adaptation and divergent female mate preferences in *Pundamilia* cichlid fish. Direct effects of visual system variation on preference could serve as a powerful mechanism of rapid ecological speciation. We found light‐induced changes in relative opsin expression, indicating that phenotypic plasticity may contribute to visual adaptation in cichlid fish. Female preference was weakly correlated with relative opsin expression, but evidence for a causal link between the two was lacking. We also found that LWS genotype covaried with female preference, when tested in broad‐spectrum light environments. Together, our results are consistent with a role of visual perception in shaping female preference for differently coloured males, but fall short of demonstrating a causal link. Moreover, our findings suggest that different components of the visual system might affect female choice in different ways. Further manipulative, QTL mapping or GWAS studies are required to elucidate these effects.

## AUTHOR CONTRIBUTIONS

MEM, OS and TGGG designed the study. RvE designed the qPCR protocol and created the standard construct. RvE, DSW and LS completed laboratory work. DSW performed the analyses, with assistance from MEM. DSW and MEM wrote the manuscript, with contributions from OS and TGGG. All authors approved the contents of this manuscript.

## Data Availability

Data and R scripts will be archived at http://www.dataverse.nl, The link for the data deposition is now available https://hdl.handle.net/10411/FKBYAY and LWS sequences will be deposited in GenBank (http://www.ncbi.nlm.nih.gov/genbank/). The Genbank accession numbers are: MN808165 ‐ MN808292.
