# Supplementary material for: Testing sensory drive speciation in cichlid fish: Linking light conditions to opsin expression, opsin genotype and female mate preference
Source: J Evol Biol. 2019 Dec 24;33(4):422–34. doi: 10.1111/jeb.13577 (PMC7187155; doi:10.1111/jeb.13577)
Supplement: Supplementary file 1 [file JEB-33-422-s001.pdf]

Supplemental Information

P. 'nyererei-like'			*Hybrid*						*P. 'pundamilia-like'*		
family	D	S	family	D	S	family	D	S	family	D	S
NN1	1	2	NP2	1	2	PN1[b]	2	3	PP1[d]	3	3
NN3[a]	3	2	NP3[1f]	1	1	PN5	1	1	PP2[d]	1	4
NN5[1b]	1	1	NP4	2	1	PN7	2	2	PP3[e]	1	3
NN6[b]	1	-	NP5	1	1	PN8[4a]	1	2	PP4[f]	0	1
NN7[1b]	1	2	NP6[2]	1	1	PN9	1	1	PP7[e]	1	4
NN14[c]	1	-	NP7[5g]	1	1	PN10[4]	1	-	PP11[3]	2	2
NN16[2c]	-	1	NP8[5g]	1	1	PN11	-	1	PP13	1	1
NN18[c]	-	1									
NN19	1	2									
NN21[5c]	-	2									
NN28[5]	1	1									
Total	*10*	*14*	*Total*	*8*	*8*	*Total*	*8*	*10*	*Total*	*9*	*18*

Table S1. Families sampled for opsin expression – Sample size for each cross, separated by family and by deep (D) and shallow (S) rearing light. Family names are expressed as mother x father, such that 'NP' indicates *P. sp. 'nyererei-like'* female x *P. sp. 'pundamilia-like'* male. NP and PN families are collectively grouped as hybrids. Superscripted numbers indicate families with the same mothers; superscripted letters indicate families with the same fathers.

SWS2b	Primer (F)	GCGCTGCACTTCCACCTC
	Primer (R)	GGCCACAGGAACACTGCAT
	Probe	FAM-TTGGATGGAGCAGGTATATCCCAGAGGG-TAMRA
SWS2a	Primer (F)	CAAGATYGAAGGTTTCATGGTA
	Primer (R)	CGCTCGAAAGCTATCACAGC
	Probe	FAM-ACTCGGTGGTATGGTAAGCCTGTGG-TAMRA
RH2A	Primer (F)	TTCTGTGCWATTGAGGATTC
	Primer(R)	CCAGGACAACAAGTGACCAGAG
	Probe	FAM-TGGCCACACTWGGAGGTGAAGTTGC-TAMRA
LWS	Primer (F)	CTGTGCTACCTTGCTGTGTGG
	Primer (R)	GCCTTCTGGGTTGACTCTGACT
	Probe	FAM-TGGCCATCCGTGCTGTTGC-TAMRA

<u>Table S2. Gene specific primers and probes</u> – Sequences of the primers/probes used in qPCR reactions.

P. 'nyererei-like'			***Hybrid***						***P. 'pundamilia-like'***		
family	D	S	family	D	S	family	D	S	family	D	S
NN3[a]	3	1	NP2	1	-	PN1[b]	-	1	PP1[d]	-	1
NN5[1b]	1	-	NP5	-	1	PN5	1	-	PP2[d]	-	1
NN6[b]	1	-				PN7	1	1	PP3[e]	-	1
NN7[1b]	1	2				PN8[4a]	-	2	PP7[e]	1	2
						PN9	1	1			
Total	*6*	*3*	*Total*	*1*	*1*	*Total*	*3*	*5*	*Total*	*1*	*5*

<u>Table S3. Females measured for both mate preference and opsin expression</u> – Of the 85 fish measured for relative opsin expression, 25 females were previously assessed for mate preference (Wright *et al*., 2017).

Table S4. LWS genotyping – LWS opsin sequences of the blue and red species and hybrids. All fish were sequenced twice, in forward (F) and reverse (R) directions and allele type was assigned from amino acid positions 216, 230, and 275 as in Seehausen et al. (2008). Heterozygous genotypes (Het) were assigned for samples with multiple nucleotide calls at polymorphic sites (see Fig. S2). *N* indicates when nucleotide identity could not be determined and question marks (?) indicate when amino acid identity could not be determined (i.e. when multiple nucleotide peaks were the same height).

P. sp. 'pundamilia-like'

family	id		nucleotide position 647	688	823	824	amino acid position 216	230	275	allele
PP2	094	F	T	A	A	T	F	T	I	P
		R	T	A	A	T	F	T	I	P
PP7	058	F	T	A	A	T	F	T	I	P
		R	T	A	A	T	F	T	I	P
PP4	883	F	T	A	A	T	F	T	I	P
		R	T	A	A	T	F	T	I	P
PP7	903	F	T	A	A	T	F	T	I	P
		R	T	A	A	T	F	T	I	P
PP3	873	F	T	A	A	T	F	T	I	P
		R	T	A	A	T	F	T	I	P
PP7	904	F	T	A	A	T	F	T	I	P
		R	T	A	A	T	F	T	I	P
PP1	833	F	T	A	A	T	F	T	I	P
		R	T	A	A	T	F	T	I	P
PP2	078	F	T	A	A	T	F	T	I	P
		R	T	A	A	T	F	T	I	P
PP4	074	F	T	A	A	T	F	T	I	P
		R	T	A	A	T	F	T	I	P
PP7	050	F	T	A	A	T	F	T	I	P
		R	T	A	A	T	F	T	I	P
PP7	070	F	T	A	A	T	F	T	I	P
		R	T	A	A	T	F	T	I	P
PP7	097	F	T	A	A	T	F	T	I	P
		R	T	A	A	T	F	T	I	P
PP2	850	F	T	A	A	T	F	T	I	P
		R	T	A	A	T	F	T	I	P
PP2	872	F	T	A	A	T	F	T	I	P
		R	T	A	A	T	F	T	I	P
PP7	880	F	T	A	A	T	F	T	I	P
		R	T	A	A	T	F	T	I	P

P. sp. 'nyererei-like'

family	id		nucleotide position 647	688	823	824	amino acid position 216	230	275	allele
NN3	053	F	A	G	T	G	Y	A	C	H
		R	A	G	T	G	Y	A	C	H
NN6	874	F	T (A)	A (G)	A (T)	G (T)	?	T	?	Het
		R	T (A)	A (G)	A (T)	G (T)	F	?	S	Het
NN3	015	F	A	G	T	G	Y	A	C	H
		R	A	G	T	G	Y	A	C	H
NN5	006	F	A	G	T	G	Y	A	C	H
		R	A	G	T	G	Y	A	C	H
NN7	017	F	T (A)	G (A)	T (A)	G (T)	?	A	?	Het
		R	T (A)	A (G)	A (T)	*N*	F	?	?	Het
NN3	047	F	A	G	T	G	Y	A	C	H
		R	A	G	T	G	Y	A	C	H
NN5	026	F	T (A)	A (G)	T (A)	G (T)	F	F	C	Het
		R	T (A)	A (G)	A (T)	T (G)	F	F	I	Het
NN5	084	F	T (A)	A (G)	T (G)	G (T)	F	T	C	Het
		R	T (A)	G (A)	A (T)	G (T)	F	A	S	Het
NN6	040	F	T (A)	A (G)	T (A)	G (T)	F	T	C	Het
		R	T (A)	G (A)	A (T)	G (T)	F	A	S	Het
NN6	045	F	A	G	T	G	Y	A	C	H
		R	A	G	T	G	Y	A	C	H
NN7	028	F	A (T)	G (A)	T (A)	G (T)	Y	A	C	Het
		R	A (T)	A (G)	A (T)	G (T)	Y	T	S	Het
NN7	085	F	A (T)	A (G)	T (A)	G (T)	Y	T	C	Het
		R	T (A)	G (A)	A (T)	G (T)	F	A	S	Het
NN5	064	F	T (A)	G (A)	A (T)	G (T)	F	A	S	Het
		R	T (A)	A (G)	A (T)	G (T)	F	T	S	Het
NN8	063	F	A	G	T	G	Y	A	C	H
		R	A	G	T	G	Y	A	C	H
NN7	843	F	A (T)	A (G)	T (A)	G (T)	Y	T	C	Het
		R	T (A)	G (A)	A (T)	T (G)	F	A	I	Het
NN3	049	F	A	G	T	G	Y	A	C	H
		R	A	G	T	G	Y	A	C	H
NN3	057	F	A	G	T	G	Y	A	C	H
		R	A	G	T	G	Y	A	C	H
NN6	067	F	T (A)	A (G)	A (T)	G (T)	F	T	S	Het
		R	T (A)	A (G)	A (T)	G (T)	F	T	S	Het
NN3	062	F	A	G	T	G	Y	A	C	H
		R	A	G	T	G	Y	A	C	H
NN8	091	F	A	G	T	G	Y	A	C	H
		R	A	G	T	G	Y	A	C	H
NN3	046	F	A	G	T	G	Y	A	C	H
		R	A	G	T	G	Y	A	C	H

Hybrid: ***P. sp. 'pundamilia-like'*** **x** ***P. sp. 'nyererei-like'***

family	id		nucleotide position 647	688	823	824	amino acid position 216	230	275	allele
PN2	099	F	T (A)	A (G)	A (T)	G (T)	F	T	S	Het
		R	T (A)	A (G)	A (T)	G (T)	F	T	S	Het
PN5	871	F	T	A	A	T	F	T	I	P
		R	T	A	A	T	F	T	I	P
PN1	065	F	T (A)	A (G)	A (T)	G (T)	F	A	C	Het
		R	T (A)	A (G)	A (T)	G (T)	F	T	S	Het
PN1	831	F	T (A)	A (G)	A (T)	G (T)	F	T	S	Het
		R	T (A)	A (G)	A (T)	G (T)	F	T	S	Het
PN9	005	F	A (T)	A (G)	A (T)	G (T)	Y	T	S	Het
		R	T (A)	A (G)	A (T)	G (T)	F	T	S	Het
PN2	909	F	A (T)	A (G)	A (T)	G (T)	Y	T	S	Het
		R	T (A)	A (G)	A (T)	G (T)	F	T	S	Het
PN5	002	F	A (T)	A (G)	A (T)	G (T)	Y	T	S	Het
		R	T (A)	A (G)	A (T)	G (T)	F	T	S	Het
PN7	918	F	T (A)	A (G)	A (T)	T (G)	F	T	S	Het
		R	T (A)	A (G)	A (T)	T (G)	F	A	S	Het
PN2	022	F	T	A	A	T	F	T	I	P
		R	T	A	A	T	F	T	I	P
PN2	075	F	T (A)	A (G)	T (A)	G (A)	F	T	C	Het
		R	T (A)	G (A)	A (T)	G (T)	F	A	S	Het
PN7	027	F	T (A)	A (G)	T (A)	G (T)	F	T	C	Het
		R	T (A)	A (G)	A (T)	G (T)	F	T	S	Het
PN2	077	F	A (T)	A (G)	A (T)	G (T)	Y	T	S	Het
		R	T (A)	A (G)	A (T)	G (T)	F	T	S	Het
PN8	082	F	A (T)	A (G)	A (T)	G (T)	Y	T	S	Het
		R	T (A)	A (G)	A (T)	G (T)	F	T	S	Het
PN8	029	F	A (T)	A (G)	A (T)	G (T)	Y	T	S	Het
		R	T (A)	A (G)	A (T)	G (T)	F	T	S	Het
PN1	025	F	T (A)	A (G)	A (T)	G (T)	F	T	S	Het
		R	T (A)	A (G)	A (T)	G (T)	F	T	S	Het
PN5	827	F	T	A	A	T	F	T	I	P
		R	T	A	A	T	F	T	I	P
PN8	055	F	T (A)	A (G)	T (A)	G (T)	F	T	C	Het
		R	A (T)	G (A)	A (T)	G (T)	Y	A	S	Het
PN2	060	F	T	A	A	T	F	T	I	P
		R	T	A	A	T	F	T	I	P

Hybrid: *P. sp. 'nyererei-like'* x *P. sp. 'pundamilia-like'*

family	id		nucleotide position 647	688	823	824	amino acid position 216	230	275	allele
NP3	894	F	A (T)	A (G)	A (T)	G (T)	Y	T	S	Het
		R	T (A)	A (G)	A (T)	G (T)	F	T	S	Het
NP2	845	F	T (A)	A (G)	T (A)	G (T)	F	T	C	Het
		R	T (A)	G (A)	A (T)	G (T)	F	A	S	Het
NP2	923	F	T (A)	A (G)	T (A)	G (T)	F	T	?	Het
		R	T (A)	A (G)	A (T)	G (T)	F	A	S	Het
NP3	061	F	T (A)	A (G)	A (T)	G (T)	F	T	S	Het
		R	T (A)	G (A)	A (T)	G (T)	F	A	S	Het
NP3	897	F	A (T)	A (G)	T (A)	G (T)	F	T	?	Het
		R	T (A)	A (G)	A (T)	G (T)	Y	?	?	Het
NP4	886	F	T (A)	A (G)	A (T)	G (T)	F	T	S	Het
		R	T (A)	G (A)	A (T)	G (T)	F	T	S	Het
NP3	033	F	T (A)	A (G)	T (A)	G (T)	F	T	C	Het
		R	A (T)	G (A)	A (T)	G (T)	Y	A	S	Het
NP4	829	F	A (T)	A (G)	A (T)	G (T)	Y	T	S	Het
		R	T (A)	A (G)	A (T)	T (G)	F	T	I	Het
NP5	030	F	A (T)	A (G)	A (T)	G (T)	Y	T	S	Het
		R	A (T)	G (A)	A (T)	T (G)	Y	A	I	Het
NP5	037	F	T (A)	A (G)	T (A)	G (T)	F	T	C	Het
		R	T (A)	A (G)	A (T)	G (T)	F	T	S	Het
NP2	882	F	A (T)	A (G)	T (A)	G (T)	Y	T	C	Het
		R	T (A)	G (A)	A (T)	G (T)	F	A	S	Het

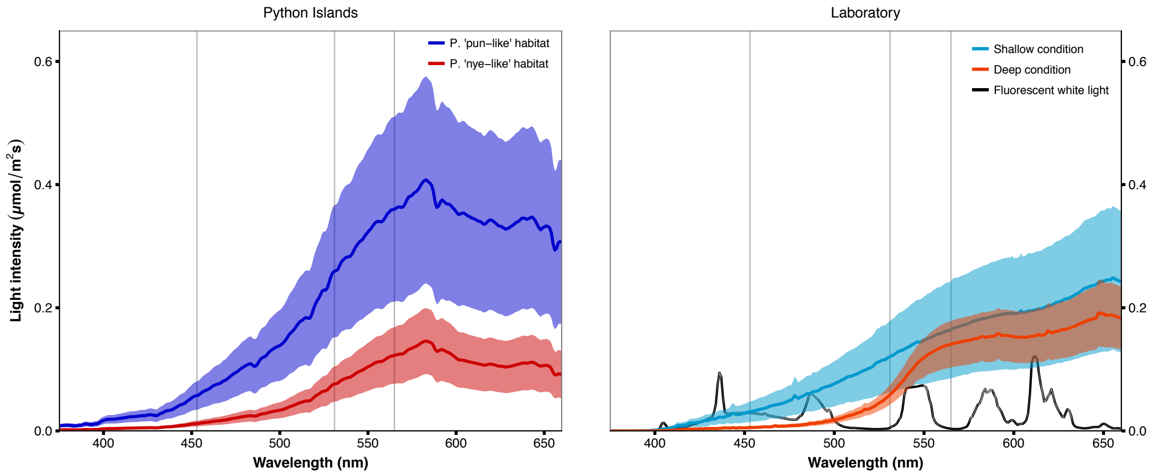

<u>Figure S1. Natural and experimental light conditions</u> – Experimental light environments were created to mimic natural light conditions experienced by *P. sp. 'pundamilia-like'* and *P. sp. 'nyererei-like'* at Python Islands, Lake Victoria. Vertical lines indicate the peak sensitivities of the three main *Pundamilia* photoreceptors: SWS2a (453nm), RH2 (531nm), LWS (565nm) (Carleton *et al.*, 2005).

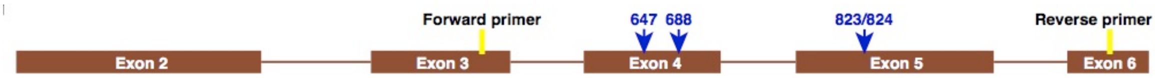

<u>Figure S2. LWS sequencing design</u> – We sequenced exons 4 and 5 of the LWS gene, to capture the known polymorphic nucleotide sites that affect the light absorption properties of the opsin (in blue). Primers were located on exons 3 and 6.

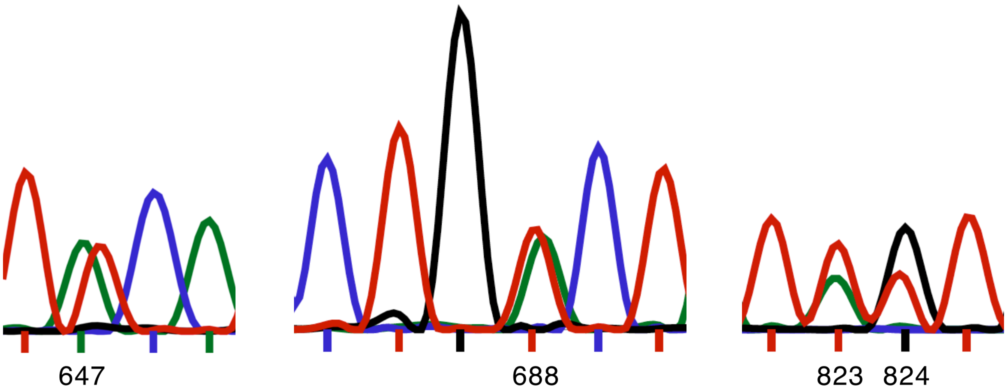

<u>Figure S3. Heterozygous LWS genotypes</u> – Sequencing results for some fish displayed multiple peaks at known polymorphic sites, shown here in an example of a sequence chromatogram for nucleotide positions 647, 688 and 823/824.

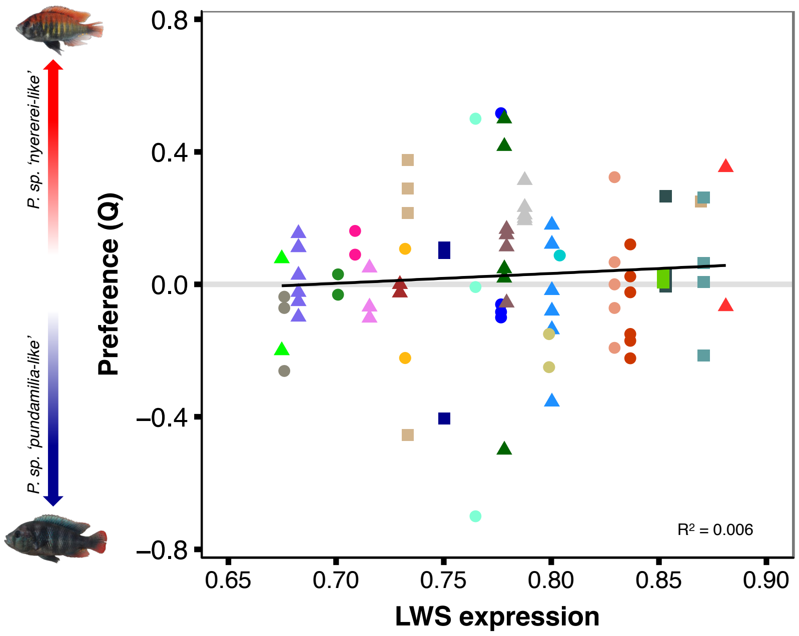

<u>Figure S4. LWS and female preference, incorporating individual replicate trials</u> - Repeatability of female preference behaviour in our prior study (Wright *et al*., 2017) was low, as indicated by the preference scores of repeatedly tested females. Colours indicate individual females (n = 25) and shapes indicate species type (▲ *P. sp. 'nyererei-like*, ● Hybrid, ■ *P. sp. 'pundamilia-like'*).

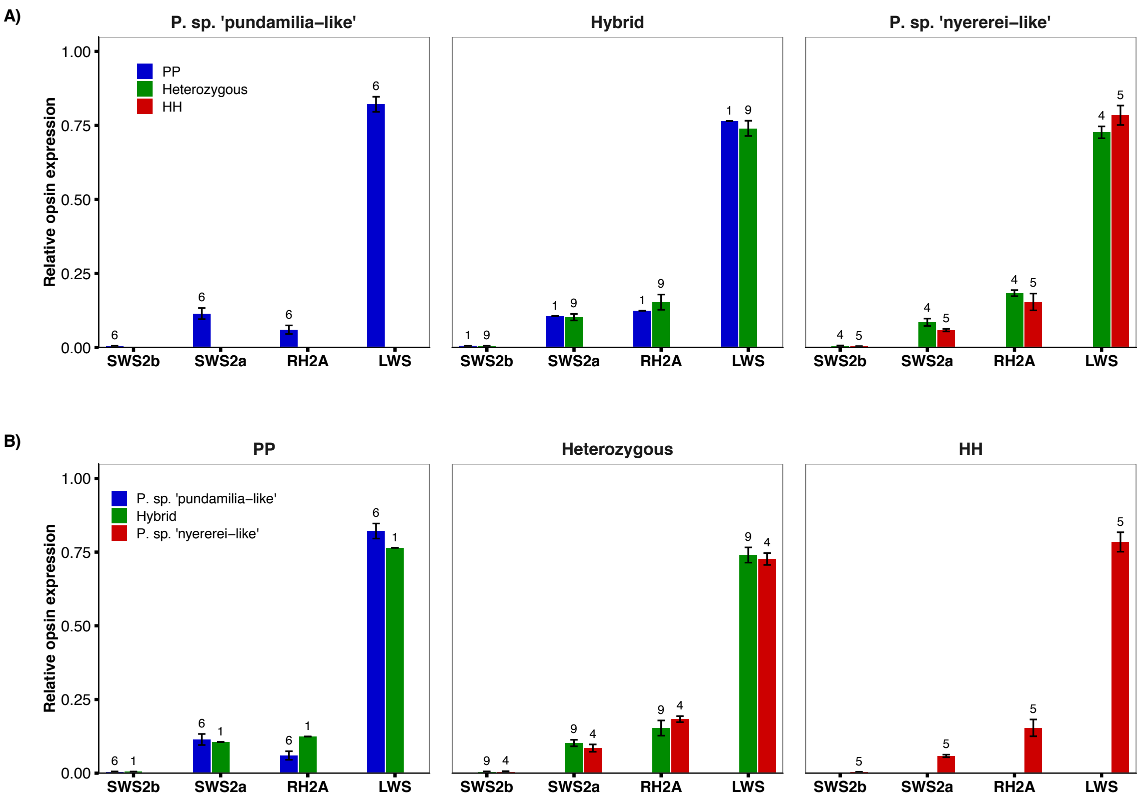

<u>Figure S5. Opsin expression profiles of LWS genotyped females</u> – Opsin expression profiles for each (A) species, coloured by genotype and each (B) LWS genotype, coloured by species. Sample sizes are indicated above each error bar and error bars represent ± standard error.

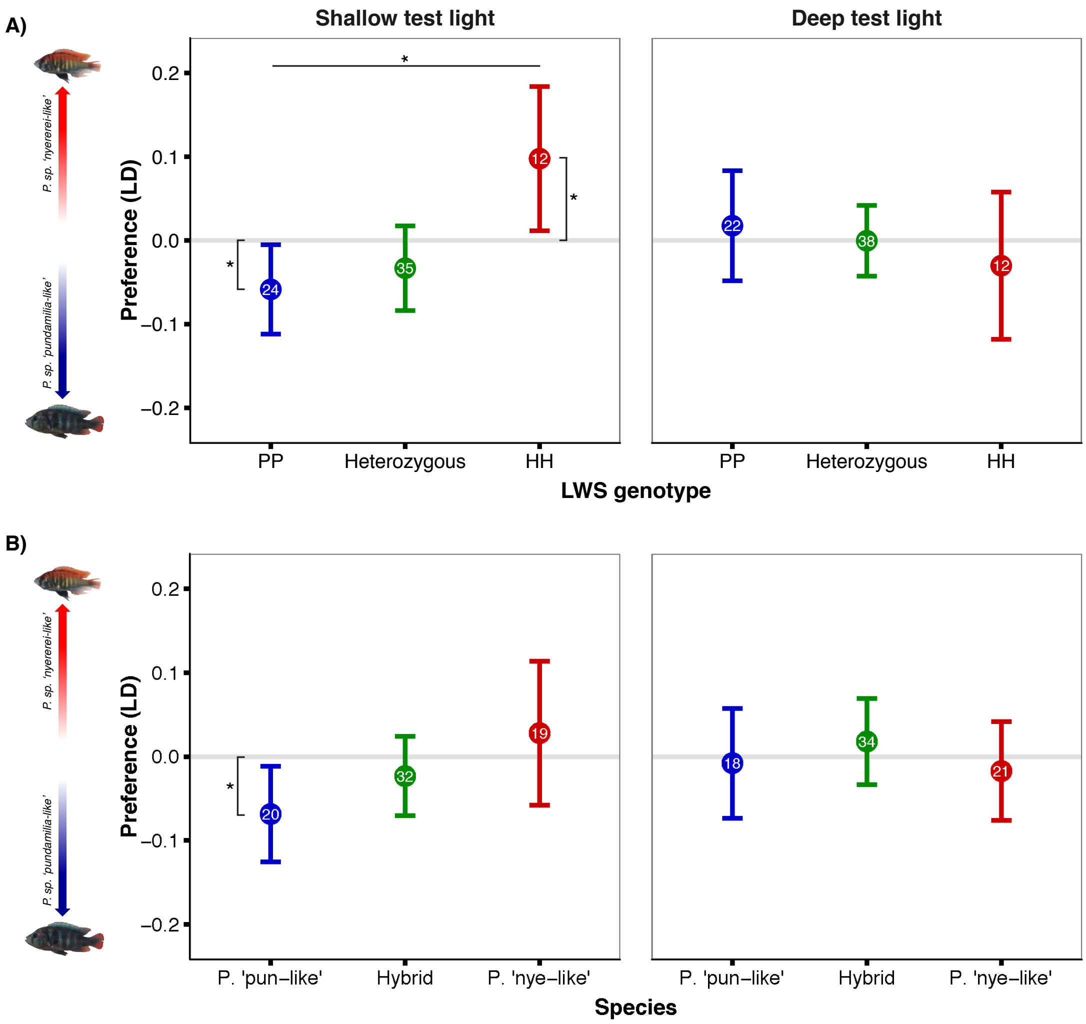

Figure S6. LWS genotype is a better predictor of female response to the light treatments –(A) There was a significant interaction between *LWS genotype* * *test light* for LD-based preference (P = 0.009; also presented in Fig. 6) but not for (B) *species* * *test light* (P > 0.15). The interaction between LWS genotype and test light suggests a role for LWS genotype in visual perception and preference determination. Colours in (A/C) indicate LWS genotype (PP, heterozygous, HH) but in (B/D) colours indicate species (*P. sp. 'pundamilia-like'*, hybrid, *P. sp. 'nyererei-like'*). Sample sizes are given within each circle and error bars represent 95% C.I. * indicates P < 0.05.

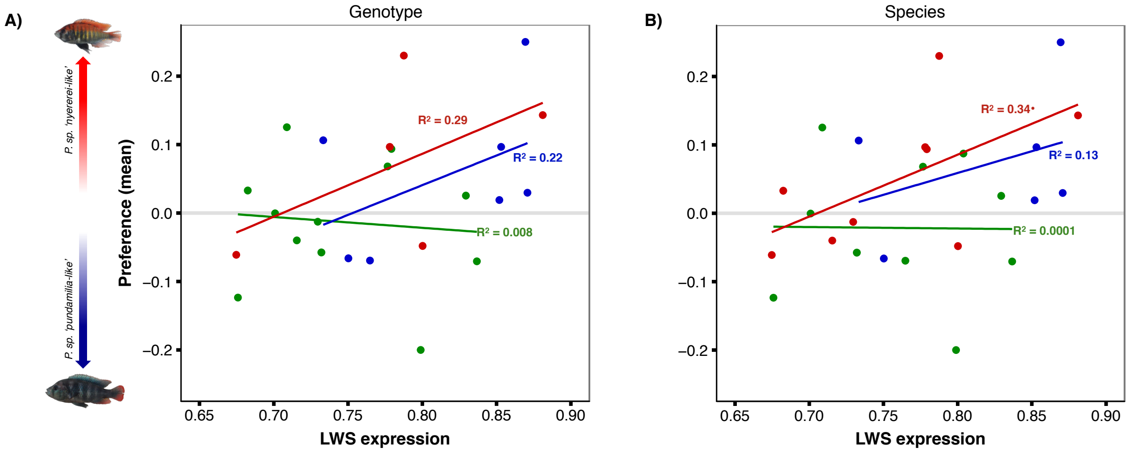

<u>Figure S7. The opsin expression-preference relationship is not influenced by LWS genotype or species identity</u> – Neither LWS genotype nor species identity better predicted the relationship between female preference and opsin expression. (A) The interaction of expression and genotype on preference was non-significant ($P > 0.4$); 'PP' and 'HH' allele types displayed similar, positive relationships between LWS expression and mate preference, while heterozygotes had a flat relationship. (B) Classifying individuals by species identity showed a very similar pattern: the interaction of expression and species identity on preference was non-significant as well ($P > 0.18$) and *P. sp. 'pundamilia-like'* / *P. sp. 'nyererei-like'* displayed similar relationships that were different from hybrids.

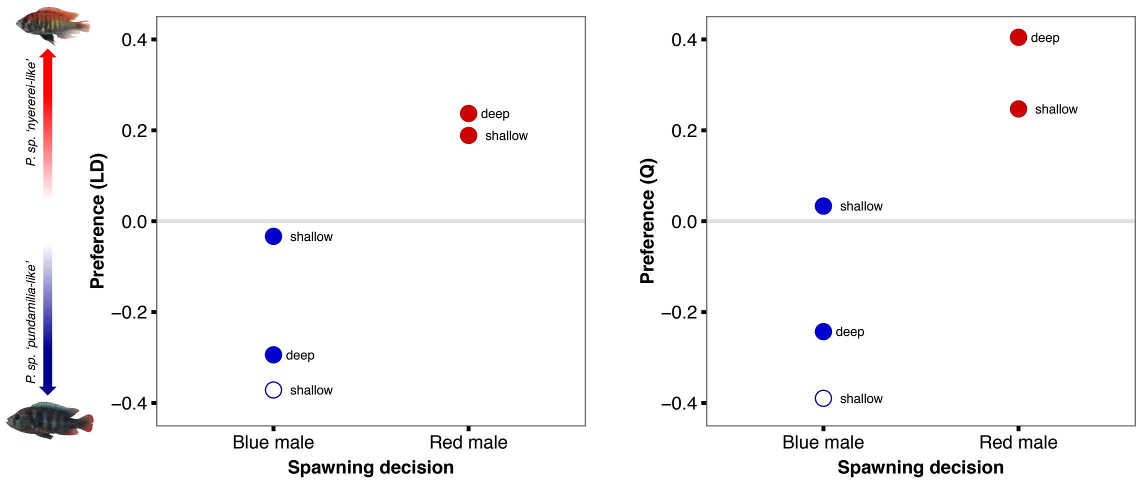

<u>Figure S8. Mate preference is a good predictor of mate choice</u> – As in our prior mate preference study (Wright *et al.*, 2017), we used a two-way preference design to assay female preference for blue vs. red males. However, we altered the partitions separating males and females to allow interaction. Males were confined behind a two-part barrier; a fixed partition made of rubber coated 'chicken wire' and a removable sheet of transparent plastic. Jointly, the two parts of the barrier mimicked the setup of the prior study - fish could see each other and exchange chemical cues but not physically interact. When the plastic divider was removed, females could enter the male compartments but males could not exit (the holes in the 'chicken wire' were too small). Thus, the setup allowed sequential testing of female mate preference and female mate choice in the same experimental trial. As before, we tested each female for preference behaviour (~20 minutes observation time) then removed the solid divider to allow the fish to mate (for the remainder of the day). Gravid females were tested in light conditions matching their rearing environment (indicated next to the circles) and female species identity was random. We tested 27 females, 5 of which spawned with one of the stimulus males (within ~8 hours). In 4 of 5 trials, females spawned with one of the two males, while in the fifth, the camera failed before mating occurred. For this trial (an open circle), we inferred the female spawning decision from the observation that the brooding female was in the compartment with the blue male (had they not spawned, we would have expected the male to be aggressively courting the female – he was not). Female choice matched preference in all cases, except one female that showed an inconsistent preference (for blue in the lateral display-based measure and for red in the quiver-based measure). These results suggest that female preference, as assessed in our setup, is a good predictor of mate choice.
